# Cardiac magnetic resonance patterns of left ventricular remodeling in patients with severe aortic stenosis referred to surgical aortic valve replacement

**DOI:** 10.1038/s41598-024-56838-0

**Published:** 2024-03-26

**Authors:** Rita Reis Santos, João Abecasis, Sérgio Maltês, Pedro Lopes, Luís Oliveira, Pedro Freitas, António Ferreira, Regina Ribeiras, Maria João Andrade, Miguel Sousa Uva, José Pedro Neves, Victor Gil, Nuno Cardim

**Affiliations:** 1 Cardiology Department, Hospital Santa Cruz, Centro Hospitalar Lisboa Ocidental, Av. Prof. Dr. Reinaldo Dos Santos, 2790-134 Lisbon, Portugal; 2 NOVA Medical School, Faculdade de Ciências da Universidade Nova de Lisboa, Lisbon, Portugal; 3 Cardiology Department, Hospital Divino Espírito Santo, Açores, Portugal; 4 Cardiac Surgery Department, Hospital Santa Cruz, Centro Hospitalar Lisboa Ocidental, Lisbon, Portugal; 5 Cardiology Department, Hospital da Luz, Lisbon, Portugal

**Keywords:** Aortic stenosis, Cardiac magnetic resonance, Left ventricular hypertrophy and remodeling, Cardiology, Valvular disease

## Abstract

Left ventricular (LV) hypertrophy is a common finding in patients with severe aortic stenosis (AS). Cardiac magnetic resonance (CMR) is the gold-standard technique to evaluate LV remodeling. Our aim was to assess the prevalence and describe the patterns of LV adaptation in AS patients before and after surgical aortic valve replacement (AVR). Prospective study of 130 consecutive patients (71y [IQR 68–77y], 48% men) with severe AS, referred for surgical AVR. Patterns of LV remodeling were assessed by CMR. Besides normal LV ventricular structure, four other patterns were considered: concentric remodeling, concentric hypertrophy, eccentric hypertrophy, and adverse remodeling. At baseline CMR study: mean LV indexed mass: 81.8 ± 26.7 g/m^2^; mean end-diastolic LV indexed volume: 85.7 ± 23.1 mL/m^2^ and median geometric remodeling ratio: 0.96 g/mL [IQR 0.82–1.08 g/mL]. LV hypertrophy occurred in 49% of subjects (concentric 44%; eccentric 5%). Both normal LV structure and concentric remodeling had a prevalence of 25% among the cohort; one patient had an adverse remodeling pattern. Asymmetric LV wall thickening was present in 55% of the patients, with predominant septal involvement. AVR was performed in 119 patients. At 3–6 months after AVR, LV remodeling changed to: normal ventricular geometry in 60%, concentric remodeling in 27%, concentric hypertrophy in 10%, eccentric hypertrophy in 3% and adverse remodeling (one patient). Indexes of AS severity, LV systolic and diastolic function and NT-proBNP were significantly different among the distinct patterns of remodeling. Several distinct patterns of LV remodelling beyond concentric hypertrophy occur in patients with classical severe AS. Asymmetric hypertrophy is a common finding and LV response after AVR is diverse.

## Introduction

Aortic valve stenosis (AS) is the most common primary valvular heart disease, with an increasing prevalence with advancing age, being an important cause of morbidity and mortality in middle aged and elderly adults^1^.

Progressive valve narrowing leads to chronic pressure increase and hemodynamic load, being the main determinant for left ventricular (LV) adaptation and remodeling^2^. However, LV response to chronic pressure overload depends also on a multitude of factors, such as age, gender, neurohormonal activation, and several comorbidities, including hypertension and reduced vascular compliance, diabetes, and chronic kidney disease^2,3^. Both structural and functional LV changes in this setting take part in the pathophysiology of the disease. Indeed, *“looking to the ventricle, beyond the valve”,* provides information toward the staging of valvular disease, with independent prognostic value for its natural history, predicting outcomes after aortic valve replacement (AVR)^4,5^.

LV remodeling is believed to begin as a compensatory mechanism to maintain wall stress. Although classically described as increased LV mass, i.e., hypertrophy, this is not always the case for LV remodeling in the context of AS^6–8^. The correlation between LV hypertrophy and the severity of valve stenosis seems to be weak and old echocardiographic studies have showed that some 10 to 20% of patients with severe AS do not have LV hypertrophy and over 10% of them have inappropriately high LV mass^6–8^. Moreover, it has been demonstrated that patients with inappropriate concentric remodelling or excessive LV mass, with the same degree of AV narrowing, are more prone to adverse cardiovascular events, both before and after AVR^6–8^.

Cardiac magnetic resonance (CMR) imaging is the gold standard technique for the assessment of LV volumes, mass, and ejection fraction^9,10^. However, most imaging studies addressing LV remodeling and adaptation in AS were performed with echocardiography, an imaging modality with inherent limitations regarding LV mass calculation and geometric assumptions. Besides that, there is paucity of information in what concerns reverse remodeling after AVR.

The aim of this study is to describe the patterns of LV adaptation and their differences both before and after surgical AVR in patients with severe AS, as assessed by CMR.

## Methods

### Study population, clinical data, and study design

Patients with isolated severe symptomatic AS (aortic valve area—AVA < 1.0 cm^2^) electively referred for surgical AVR at our tertiary single-centre, between 2019 and 2022, were prospectively included in the study. This is part of a research protocol involving both pre- and post-operative LV structural and functional assessment by multimodality imaging. Study approval was granted by the ethical committee of Nova Medical School University (number 61/2018/CEFCM) conforming to the principles of the Helsinki declaration. All participants gave written informed consent before inclusion.

Patients with previous diagnosis of sub/supra valvular aortic stenosis, concomitant severe non-aortic valve dysfunction and associated moderate to severe aortic regurgitation were excluded. Those with any of the following conditions were also excluded: previous cardiac surgery, active endocarditis, previous history of myocardial infarction, myocarditis, ischemic and non-ischemic cardiomyopathy including amyloidosis and other infiltrative diseases, non-cardiac inflammatory disease, active infection, under immunosuppressive and chronic anti-inflammatory therapy, under chemotherapy and with previous chest radiotherapy.

Clinical parameters (demographics, major cardiovascular risk factors, symptomatic status including the presence of angina, syncope, and New York Heart Association [NYHA] class, current medication) were collected at the time of inclusion, which was followed, at the same day, by both twelve-lead electrocardiography (ECG) and transthoracic echocardiography (TTE). Cardiac magnetic resonance (CMR) ensued at the same day or at no more than 2 weeks after patient inclusion, and a blood sample was collected at the day of this examination for the specific evaluation of hematocrit (Htc), renal function (creatinine, urea), high sensitivity cardiac troponin T (hsTnT) and N-terminal pro b-type natriuretic peptide (NT-ProBNP). Both TTE and CMR studies were planned to be performed within 6 months before AVR.

If clinically justified for coronary artery disease exclusion, patients performed a coronary angiography before intervention, and coronary revascularization was added to AVR when indicated. Surgical myectomy was concomitantly performed if planned before intervention because of asymmetric septal hypertrophy or at surgeon´s discretion. As per institutional protocol all patients underwent pre-discharge TTE following surgery, whose data were not included in the analysis.

To assess LV morphological and functional changes after surgery, all patients had a detailed TTE and CMR study between the 3^rd^ and 6^th^ month after surgery, as there is evidence that the majority (> 80%) of volumetric and geometric LV changes have already occurred at this time point^11^. These studies included the same parameters as that from pre-intervention.

### Standard echocardiographic study—evaluation for aortic stenosis

All patients underwent a comprehensive TTE by experienced cardiologists before AVR, using commercially available ultrasound systems (Vivid E95; GE Healthcare, Chicago, IL, USA) with a 4D probe (3.5-MHz 2D phased array transducer), in accordance with current guidelines^12,13^. Imaging analysis and measurements were performed on image data stored in the image vault and re-examined using EchoPAC version 202 for PC (GE Healthcare, Milwaukee, WI, USA). As proposed^12,14^ and for the quantification of AS, LV outflow tract diameter was measured on parasternal long-axis view and pulsed-wave and continuous-wave Doppler were used to record velocities across the LV outflow tract and aortic valve (AV), respectively. LV stroke volume index was calculated as LV outflow tract velocity time integral (obtained at 5-chamber apical view) x LV outflow area/body surface area. Multiple TTE windows, including apical, subcostal, right parasternal and suprasternal, were assessed to obtain the highest well defined AV velocity signal, which was used to obtain the peak AV velocity and mean AV gradient, estimated using the Bernoulli equation. AV area was calculated with the continuity equation.

In patients with low gradient AS, measurements of aortic valve area were checked for correctness. Both aortic valve calcium and low dose dobutamine were used according to LVEF and flow status, in accordance with the guidelines.

All reported bidimensional and Doppler derived measurements were averaged over 3, or at least 5 cardiac cycles for patients in atrial fibrillation. Additional echocardiographic evaluation, beyond aortic valve assessment, was performed in all patients as a comprehensive TTE, in accordance with current guidelines^13,15^.

TTE study at the 3–6th month after AVR included all the above-mentioned parameters in addition to specific prosthetic assessment, as recommended^16^. This involved peak velocity and mean transprosthetic gradient, acceleration time at the contour of the jet velocity, Doppler velocity index and the effective orifice area (EOA) when in the presence of above-normal prosthetic gradients. Moderate and severe patient-prosthesis mismatch was defined as indexed EOA below 0.85cm^2^/m^2^ and 0.65cm^2^/m^2^, respectively.

### Cardiac magnetic resonance

CMR study was performed at a 1.5 Tesla scanner (*Magnetom Avanto; Siemens Medical Solutions, Erlangen, Germany*), using the protocol described by *Kramer CM. *et al.^17^. Post-processing and quantification were performed using a dedicated software (*Circle Cardiovascular Imaging, CVI version 5.12, Calgary, Canada*). LV volume, mass and ejection fraction were measured using standard volumetric techniques after endocardial and epicardial delineations in all end-diastolic and end-systolic phase short-axis images. Native and postcontrast T1 mapping was performed using a *Modified Look-Locker Inversion* recovery (MOLLI) sequence in expiratory apnea, into three segments of the LV short-axis (base, mid and apex) before and 15 to 20 min after contrast injection, for extracellular volume (ECV) quantification, defined as ECV = (1-Htc) × [ΔR1 myocardium]/[ΔR1 blood]^18^, with the estimated hematocrit (Htc) from the collected blood sample at the same day. The LV short-axis stack of LGE was first assessed visually for the presence of LGE, followed by its quantification, when present, also after endocardial and epicardial delineations. LGE was defined as areas of signal intensity ≥ 5 standard deviations from normal myocardium and was expressed as total mass and the percentage of total LV myocardial mass. Those with subendocardial LGE were excluded from the analyses.

LV mass and volumes were indexed to body surface area (calculated using the *Mosteller* formula). Left ventricular dilatation and hypertrophy were defined as a higher-than-normal left ventricular indexed end-diastolic volume (LVEDVi) and mass (LVMi), respectively, according to age and gender^19^. Geometric remodelling was calculated by dividing the LV mass by the left ventricular end-diastolic volume, being used as an indicator of LVMi normalized to chamber size^20^.

All patients, including those in atrial fibrillation, had controlled heart rates (between 50 and 90 beats per minute). All CMR results were read by two experienced readers (JA and AF) with Level 3 CMR accreditation by the European Society of Cardiovascular Imaging, blinded to both clinical and echocardiographic data.

### Patterns of LV hypertrophy and remodelling—definitions

The adaptation of the LV to chronic pressure overload and AS has been previously defined by the combined assessment of LV mass, cavity dimensions/volumes and wall thickness^21,22^.

As previously proposed by Dweck et al.^9^, five patterns of LV adaptation were defined in our cohort, as assessed by both pre and post-operative CMR and according to the LVMi, LVEDVi and M/V ratio (Supplementary table 1): normal LV ventricular structure, concentric remodeling concentric hypertrophy, eccentric hypertrophy, and adverse remodeling. Asymmetric LV hypertrophy was also considered, regardless of the other five patterns, and defined as a regional wall thickening ≥ 13 mm (from the short-axis views of the LV in end diastole, with exclusion of LV trabeculations) that was also > 1.5-fold greater than the thickness of the opposing myocardial segments, on at-least two adjacent short-axis slices.

### Statistical analysis

Categorical values are presented as absolute numbers (and percentage). Continuous variables as mean ± standard deviation (normal distribution) or as median and interquartile range (IQR; non-parametric). Kolmogorov–Smirnov test was used to test normality of the variables.

Independent and paired sample T-Test, Mann–Whitney U, Wilcoxon, One-way ANOVA and Pearson’s Chi-squared (x^2^) tests were applied for comparison where appropriate. Statistical significance was set at P-value < 0.05 (two-sided). Correlation between normally distributed data was performed using Pearson’s correlation to provide r^2^ values. To assess correlations between NT-proBNP and late gadolinium enhancement (LGE) mass with LVMi, Spearman correlation coefficients were used. All analyses were performed using Statistical Package for the Social Sciences Statistics v27.0 (IBM Corporation, Armonk, NY, USA).

### Ethics approval

This study was approved by the local ethics committee and the ethical committee of Nova Medical School University (number 61/2018/CEFCM), fulfilling the principles of the Helsinki declaration.

### Consent to participate

All patients gave written informed consent before pre-operative imaging studies.

## Results

### Study population and clinical data

Overall, we prospectively studied 130 consecutive patients with severe symptomatic AS and complete pre-operative CMR study, with an age of 71 years [IQR 68–77y], 62 (48%) men. A mean period of 3.9 ± 2.5 months elapsed between imaging studies and valve surgery. Demographic, clinical and laboratory related data are summarized in Table 1. Regarding cardiovascular risk factors and comorbidities, at pre-operative study: 89% (n = 116) had a previous diagnosis of hypertension; 26% (n = 34) patients were diabetic, and 32% of patients (n = 42) had chronic kidney disease defined by a glomerular filtration rate below 60 mL/min/1.73m^2^ (all of them with creatinine clearance above 25 ml/min; no patient under dialysis). All patients with hypertension were under therapy with at least one anti-hypertensive agent (predominantly with ACE inhibitors/ARBs: 65%; followed by diuretics: 36%; beta-blockers 29%; and 2% with MRAs). The specific assessment of appropriate blood pressure control before surgery, such as ambulatory measurements, was not performed.

**Table 1:** Baseline clinical, laboratory and surgical data of the study cohort.

Total study population: n = 130	
Clinical characteristics	
Age, years	71 (68–77)
Male	62 (47.7%)
BSA m^2^	1.80 ± 0.19
Hypertension	108 (83%)
Diabetes mellitus	34 (26.1%)
Dyslipidemia	78 (60%)
Chronic Kidney disease	42 (32.3%)
NYHA functional class	
I	7 (5.4%)
II	99 (76.2%)
III	24 (18.5%)
Anginal symptoms	35 (26.9%)
Syncope	30 (23.1%)
Laboratory results	
Creatinine, mg/dL	0.91 (0.77–1.11)
Estimated glomerular filtration rate, mL/min	70.1 (55.5–86.9)
NT-proBNP, pg/mL	568 (219–1479)
Troponin, ng/L	13 (9–20)

Values are median (interquartile range), mean ± standard deviation.

*BSA* body surface area, *NYHA* New York Heart Association.

### Preoperative imaging evaluation (Table 2)

**Table 2:** Baseline imaging data: TTE and CMR.

Total study population: n = 130	
Echocardiography	
Aortic valve area, cm^2^	0.7 ± 0.2
Maximum aortic gradient, mmHg	98.6 ± 27.2
Mean aortic gradient, mmHg	61.1 ± 17.6
Stroke volume index, mL/m^2^	47.5 ± 10.9
Relative wall thickness	0.51 ± 0.13
Septal thickness, mm	16 ± 2.7
LV mass, g	287.8 ± 98.3
LV indexed mass, g/m^2^	158.3 ± 53.2
LVEDV, mL	86.9 ± 33.9
LVEDV indexed, mL/m^2^	48.1 ± 17.1
LVESV, mL	41.7 ± 21.9
LVESV indexed, mL/m^2^	22.9 ± 11.1
LV ejection fraction, %	57.9 ± 9.3
Global longitudinal strain, %	− 14.7 ± 3.8
Cardiac magnetic resonance (CMR)	
LV mass, g	147.9 ± 52.7
LV indexed mass, g/m^2^	81.8 ± 26.7
LVEDV, mL	155.1 ± 47.4
LVEDV indexed, mL/m^2^	85.7 ± 23.0
LVESV, mL	65.3 ± 33.6
LVESV indexed, mL/m^2^	36.1 ± 17.4
Geometric remodeling, g/mL	0.96 (0.82–1.08)
LV ejection fraction, %	59.7 ± 10.1
Global native T1, ms	1053 (1023–1071)
Global ECV, %	24 (21–27)

Values are median (interquartile range), mean ± standard deviation.

*ECV* extracellular volume, *LV* left ventricle, *LVEDV* left ventricle end diastolic volume, *LVESV* left ventricle end systolic volume.

Mean aortic valve area was 0.7 ± 0.2 cm^2^ and 96% of patients had high gradient (> 40 mmHg) AS (mean transvalvular pressure gradient of 61 ± 18 mmHg). Five patients had a mean transvalvular gradient between 35 and 40 mmHg. Normal flow condition (stroke volume index above 35 mL/m^2^) occurred in 85% (n = 110) of patients. Mean LVEF by TTE was 58 ± 9% and mean global longitudinal strain was − 14.7 ± 3.8%. Only 7 patients (5%) had preoperative LVEF below 40% (85% with LVEF > 50%; 10% with LVEF between 40 and 50%). Overall, the great majority of patients (n = 107; 82%) had classical normal flow, high gradient severe AS. Low-flow low-gradient aortic stenosis, defined as: mean transaortic gradient < 40 mmHg, valve area < 1.0cm^2^ and SVi < 35 mL/m^2^, was present in 2 patients.

Mean LV indexed mass before surgery at the TTE was 158.1 ± 53.2 g/m^2^, with a mean relative wall thickness (RWT) of 0.5 ± 0.13.

At preoperative CMR study, mean LVEF was 60 ± 10%, mean indexed LV mass [LVMi]: 81.8 ± 26.7 g/m^2^; indexed mean end-diastolic LV volume [LVEDVi]: 85.7 ± 23.1 mL/m^2^ and median geometric remodeling ratio [M/V]: 0.96 g/mL [IQR 0.82–1.08 g/mL]. Non-ischemic late gadolinium enhancement (LGE), excluding patients with exclusive junctional LGE, was observed in 70 patients (54%), all with a non-ischemic combined mid-wall and junctional enhancement pattern, except one patient who presented a non-transmural subendocardial LGE (previously unknown ischemic scar).

### Patterns of left ventricular adaptation before surgery

**Figure 1:**
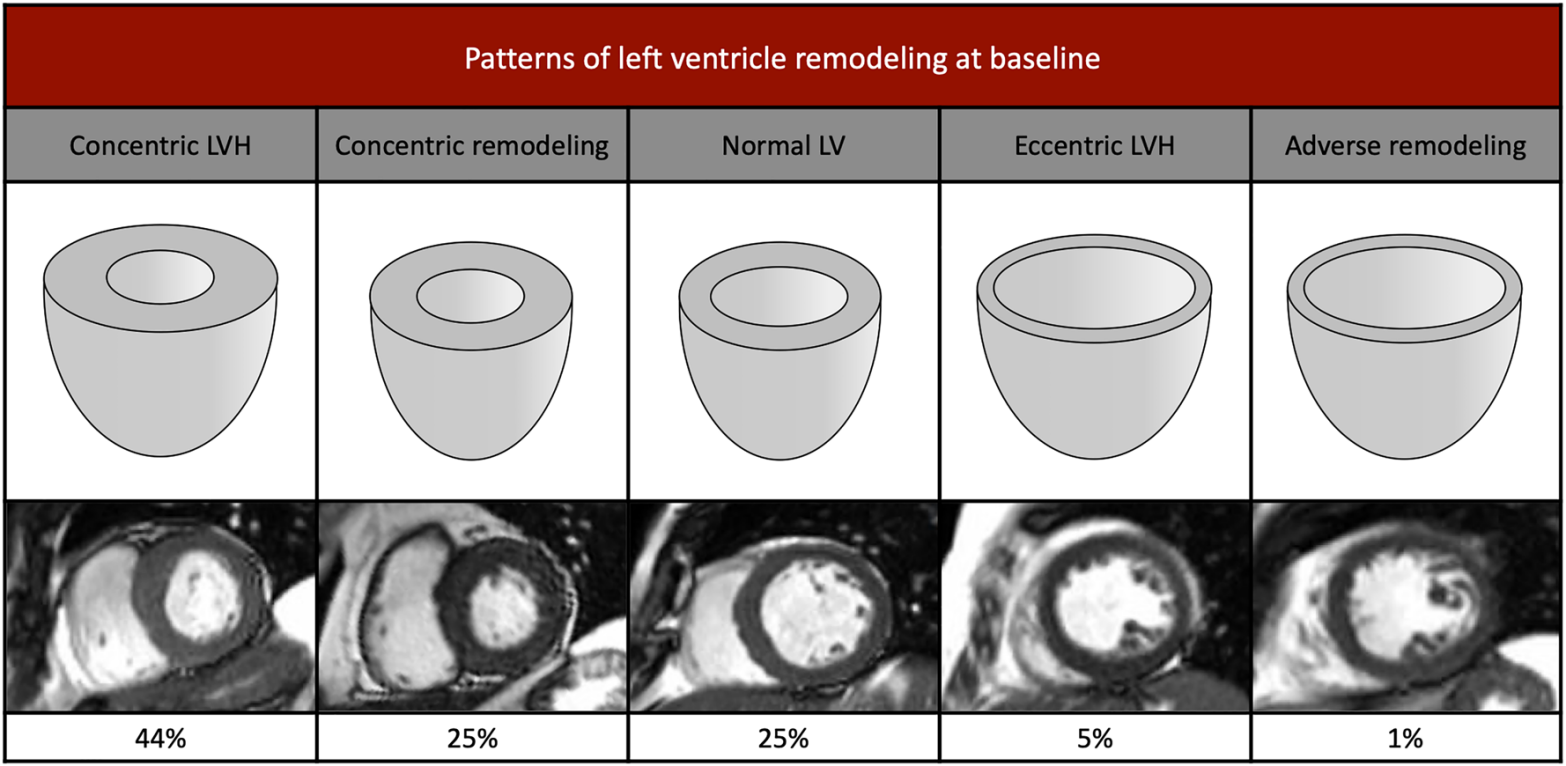
Patterns of left ventricle remodeling at baseline/preoperative assessment—left ventricular structure alongside CMR short-axis images.

Concentric hypertrophy and remodeling were the predominant patterns of LV adaptation (representing 69% of the patients). Notably, 25% of the patients had normal LV geometry before surgery (Fig. 1) and LV wall asymmetry was present in 71 (55%) patients, most often affecting the basal anterior and mid inferior septum. None of these patients had significant pre-operative intra-ventricular gradients. All patients with reduced LVEF except one had normal LV volumes (only one patient matching the definition of adverse remodeling).

Meaningful differences were observed regarding AS severity, echocardiographic parameters of LV function (LVEF and GLS) and loading (E/E´, Left atrial indexed volume, NT-ProBNP values) when comparing patients with distinct patterns of LV adaptation (Table 3). LVEF (as assessed by CMR) was also distinct between different patterns of LV remodelling. At tissue characterization, patients with concentric hypertrophy had higher absolute masses of LGE.

**Table 3:** Comparative analyses between different patterns of LV structure in patients with severe symptomatic AS before AVR.

	Normal ventricle	Concentric remodeling	Concentric hypertrophy	Eccentric hypertrophy	Adverse remodeling	*p* value*
Number of patients	33	33	57	6	1	–
Male sex, %	35	53	51	17	100	0.144
Age, years	72 ± 9	73 ± 7	70 ± 8	73.8 ± 7.6	67	0.591
Hypertension, n (%)	28 (85)	26 (79)	47 (82)	6 (100)	1 (100)	0.521
ACE inhibitors/ARBs, n (%)	24 (73)	17 (52)	38 (67)	5 (83)	0	0.176
Diuretics, n (%)	15 (46)	8 (24)	21 (37)	3 (50)	0	0.337
Beta-blockers, n (%)	13 (39)	12 (36)	10 (18)	3 (50)	0	0.058
MRAs, n (%)	1 (3)	0	2 (4)	0	0	0.826
Diabetes mellitus, n (%)	5 (15)	10 (30)	17 (30)	2 (33)	0	0.527
Dyslipidemia, n (%)	18 (55)	23 (70)	32 (56)	5 (83)	0	0.301
Creatinine Clearance, ml/min	73.5 ± 22.7	66.6 ± 21	74.7 ± 23.8	63.6 ± 26	97.8	0.307
NT-pro BNP, pg/mL	417.5 (181.2–1188.3)	219.1 (131–426.1)	954 (475–2686)	1709 (1201.3–3019.3)	7493	** < 0.001**
Echocardiography						
Indexed Aortic Valve Area, cm^2^	0.40 ± 0.2	0.43 ± 0.07	0.38 ± 0.10	0.43 ± 0.67	0.44	0.080
Mean Aortic gradient, mmHg	54.0 ± 15.3	51.9 ± 8.8	71.1 ± 17.6	55.9 ± 13.3	50.1	**0.001**
Stroke volume index, mL/m^2^	44.8 ± 8.7	45.9 ± 9.0	49.5 ± 12.8	51.1 ± 10.7	48.6	0.284
Relative wall thickness	0.48 ± 0.13	0.54 ± 0.14	0.53 ± 0.12	0.40 ± 0.15	0.31	**0.012**
LV indexed mass, g/m^2^	138.7 ± 44.3	128.9 ± 22.6	183.3 ± 58.1	186.9 ± 54.1	177.0	** < 0.001**
Indexed LVEDV, mL/m^2^	40.5 ± 16.9	34.1 ± 11.7	53.3 ± 19.0	67.0 ± 7.1	89.4	** < 0.001**
LV ejection fraction, %	60.6 ± 6.4	59.7 ± 9.3	56.4 ± 9.8	53.7 ± 7	35	**0.012**
GLS, %	− 17.3 ± 3.3	− 15.8 ± 3.6	− 12.9 ± 3.4	− 14.6 ± 4	− 11.6	** < 0.001**
E/A ratio	0.77 (0.61–1.14)	0.74 (0.56 – 0.78)	0.71 (0.56–0.89)	1.6 (0.72–1.7)	0.66	0.363
E/e’	11.1 (8.9–17.1)	12.0 (9.7–17)	14.5 (12–20.5)	26.5 (13.5–32.5)	13.6	**0.013**
LAVi (ml/m2)	39.2 (35–49.5)	36.4 (27,5 – 42.9)	44.3 (36.3–57.5)	58.2 (48.5–72.3)	42.1	** < 0.001**
PASP (mmHg)	38 (28.8–49.0)	37 (31–37)	34 (29–50)	48 (39–59)	45	0.522
TAPSE, mm	22.1 ± 3.3	22.5 ± 3.9	21.5 ± 3.9	21.2 ± 4.6	29	0.262
STDI, cm/s	11.2 ± 6.7	8.2 ± 7.3	11.3 ± 5.6	8.7 ± 7.4	14	0.194
Cardiac magnetic resonance						
Ejection fraction, %	61.3 ± 8.2	63.9 ± 9.2	56.8 ± 10.7	57.9 ± 7.4	41	**0.003**
LV mass index, g/m^2^	59.8 ± 10.6	66.7 ± 10.2	103.1 ± 24.7	81.9 ± 15.9	89.9	** < 0.001**
Indexed LVEDV, mL/m^2^	80.5 ± 15.4	66.7 ± 11.2	96.6 ± 23.4	106.9 ± 12.1	139.7	** < 0.001**
Geometric remodeling, g/mL	0.75 (0.68–0.82)	0.98 (0.92–1.09)	1.03(0.95–1.17)	0.75 (0.70–0.82)	0.64	** < 0.001**
Asymmetric hypertrophy, n (%)	11 (33)	15 (50)	42 (74)	3 (50)	0	** < 0.001**
LGE, g	0 (0–5.55)	3.63 (0–6.65)	6.2 (1.67–13.3)	0.27 (0–5.64)	2.70	0.013
LGE, % of mass	0 (0–4.55)	3.35 (10–6.53)	4.5 (1.04–8.05)	0.25 (0–4.10)	1.60	0.041
Global native T1, ms	1056 (1013–1074)	1041 (1018–1062)	1056 (1036–1074)	1063 (1039–1078)	1070	0.210
Global ECV, %	25 (21–28)	24 (21–26)	23 (19–27)	21 (20–23)	28	0.238

Values are median (interquartile range), mean ± standard deviation.

*LV* left ventricle, *LVEDV* left ventricle end diastolic volume, *LAVI* left atrium volume index, *PASP* pulmonary artery systolic pressure, *TAPSE* tricuspid annulus plane systolic excursion, *STDI* right ventricular systolic velocity at tissue Doppler imaging, *ECV* extracellular volume, *LGE* late gadolinium enhancement.

*p value provided from comparisons between 5 remodeling LV patterns according to one-way ANOVA; Bold *p-*values are statistically significant; Patients with exclusive junctional LGE were excluded from the analyses of LGE; LGE quantification was performed in 70 patients.

No difference was observed regarding the prevalence of hypertension, creatinine clearance and use of different anti-hypertensive classes of drugs.

When assessing those with asymmetric LV wall thickening, these patients had higher transvalvular gradients and NT-proBNP levels. At CMR, this group was characterized by lower LVEF, higher LVMi and absolute LGE mass (Supplemental Table 2). Prevalence of asymmetric hypertrophy was significantly higher in patients with concentric hypertrophy pattern (Table 3; Supplemental Table 2). There were no significant differences among distinct patterns of remodeling and LV asymmetric hypertrophy regarding native T1 myocardium values and ECV. From the multiparametric pre-operative CMR study involving both mapping derived indexes and LGE, there was no suspicion of amyloid infiltration at the individual patient level.

**Figure 2:**
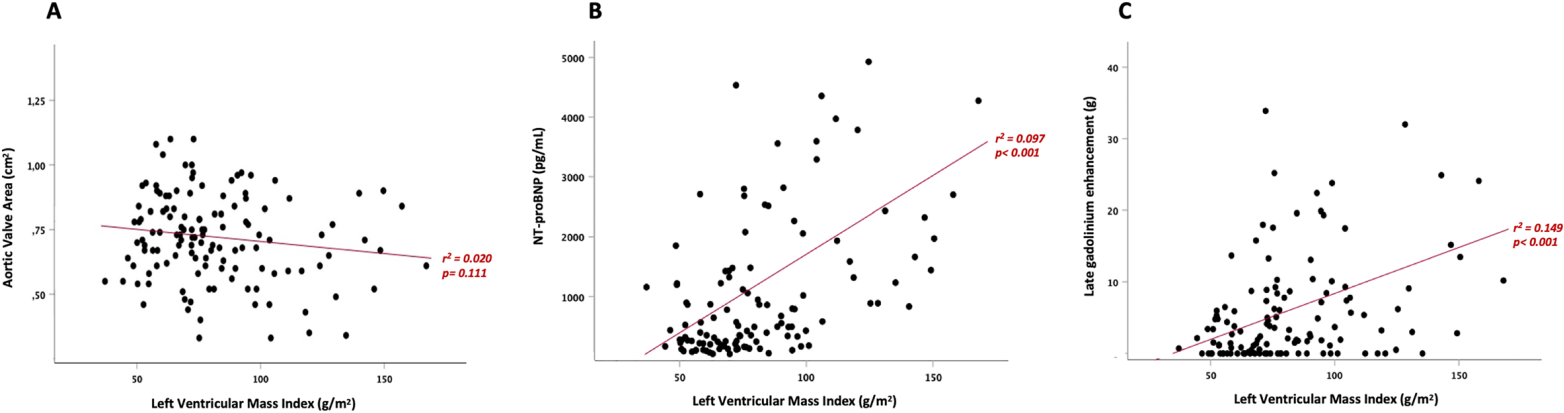
Correlation analyses: lack of correlation between aortic valve area and left ventricular mass index (LVMi) by CMR; Significant correlations between LVMi (by CMR) and NT-proBNP and LVMi (by CMR) and late gadolinium enhancement.

No significant correlation was found between LVMi and aortic valve narrowing. However, LV mass was significantly related to both NT-proBNP levels and LGE mass (Fig. 2).

### Evolution of LV remodeling after AVR

AVR was already performed in 119 patients (92%) and 89% (116 patients) completed the post-operative study with TTE and CMR at the 3^rd^ to 6^th^ month post-AVR. Comparative analysis between pre- and postoperative imaging data at both TTE and CMR studies revealed significant reductions of LV mass and volumes after surgery, with significant improvement of LV systolic function indexes (Table 4).

**Table 4:** Comparative analyses between imaging data: TTE and CMR in patients with severe symptomatic AS before and at the 3rd to 6th month post-surgical AVR.

	Before AVR n = 130	After AVR n = 116	*p *value
Echocardiography			
Maximum aortic gradient, mmHg	98.6 ± 27.2	23.1 ± 8.7	**< 0.001**
Mean aortic gradient, mmHg	61.1 ± 17.6	12.7 ± 5.2	**< 0.001**
DVI	0.20 ± 0.05	0.49 ± 0.10	**< 0.001**
Relative wall thickness	0.51 ± 0.13	0.46 ± 0.14	**0.001**
LV indexed mass, g/m^2^	158.3 ± 53.2	128.9 ± 44.2	**< 0.001**
LVEDV, mL	86.9 ± 33.9	85.5 ± 32.7	0.607
LV ejection fraction, %	57.9 ± 9.3	59.7 ± 7.9	0.095
Global Longitudinal strain, %	− 14.7 ± 3.8	− 16.2 ± 3.2	**< 0.001**
Cardiac magnetic resonance			
LV indexed mass, g/m^2^	81.8 ± 26.7	62.7 ± 15.1	**< 0.001**
LVEDV, mL	155.1 ± 47.4	141 ± 44.2	**< 0.001**
Geometric remodeling, g/mL	0.96 (0.82–1.08)	0.84 (0.71–0.93)	**< 0.001**
LV ejection fraction, %	59.7 ± 10.1	58 ± 8.8	**0.034**

Values are median (interquartile range), mean ± standard deviation.

*LV* left ventricle, *LVEDV* left ventricle end diastolic volume.

Bold *P* values are statistically significant. Comparative sensitivity analysis was performed only for patients who complete post-operative study with TTE and CMR at the 3rd to 6th month post-AVR.

There was no severe prosthesis dysfunction at echocardiographic follow-up. Two patients with above labeled prosthetic gradients were identified with moderate patient-prosthesis mismatch. Four of 116 patients (3%) had mild paravalvular regurgitation. None of the patients presented intraventricular or left ventricular outflow tract gradients at TTE follow-up after AVR. The majority of patients (94%) had a bioprosthetic implantation and subaortic septal myectomy was added to the procedure, as reported by the surgical team, in 60% (n = 71) of the cases. At surgical report there was no reference to the extension of myectomy, namely in what concerned the dimension of the excised sample of the interventricular septum.

**Figure 3:**
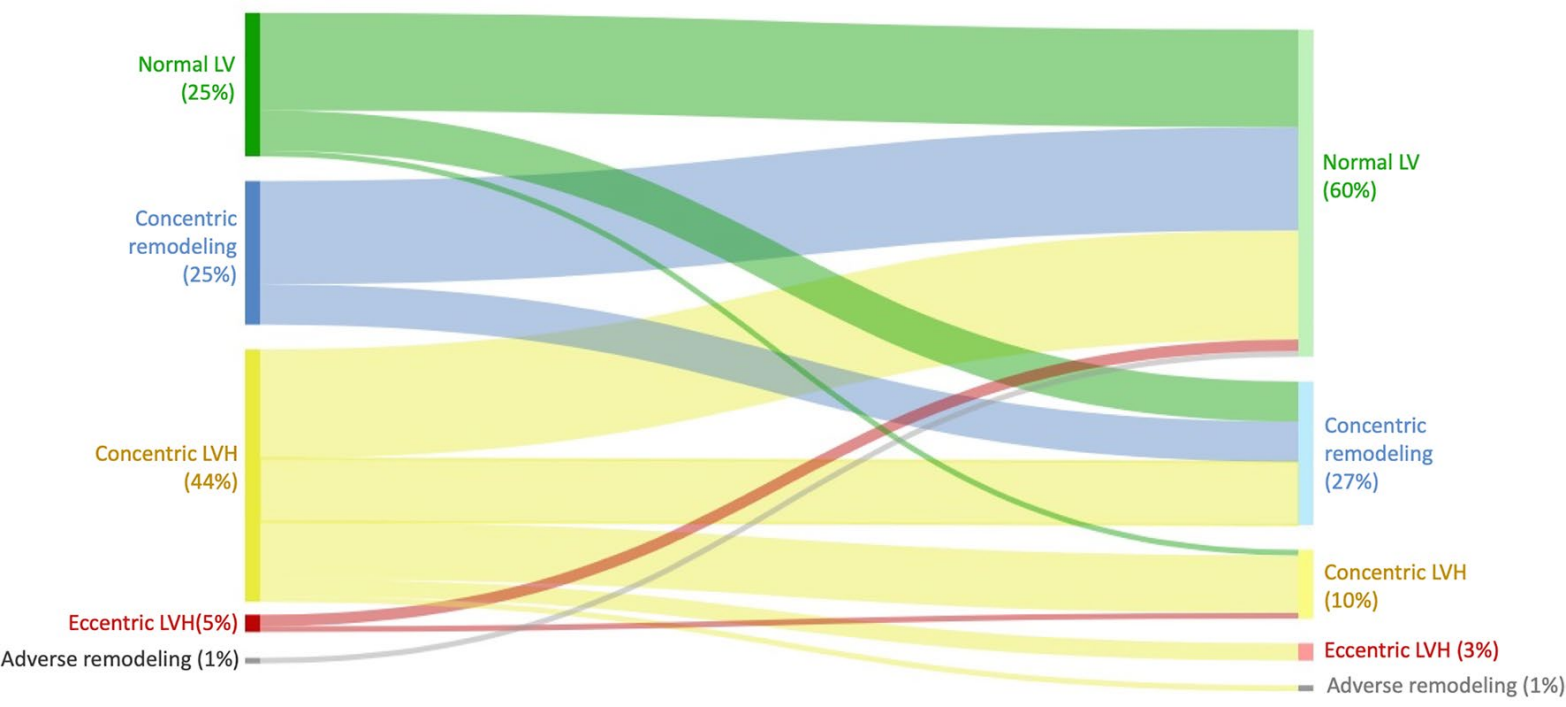
*Sankey* diagram of cardiac magnetic resonance patterns (pre- and post-surgical aortic valve replacement) of left ventricular hypertrophy in aortic stenosis patients.

**Figure 4:**
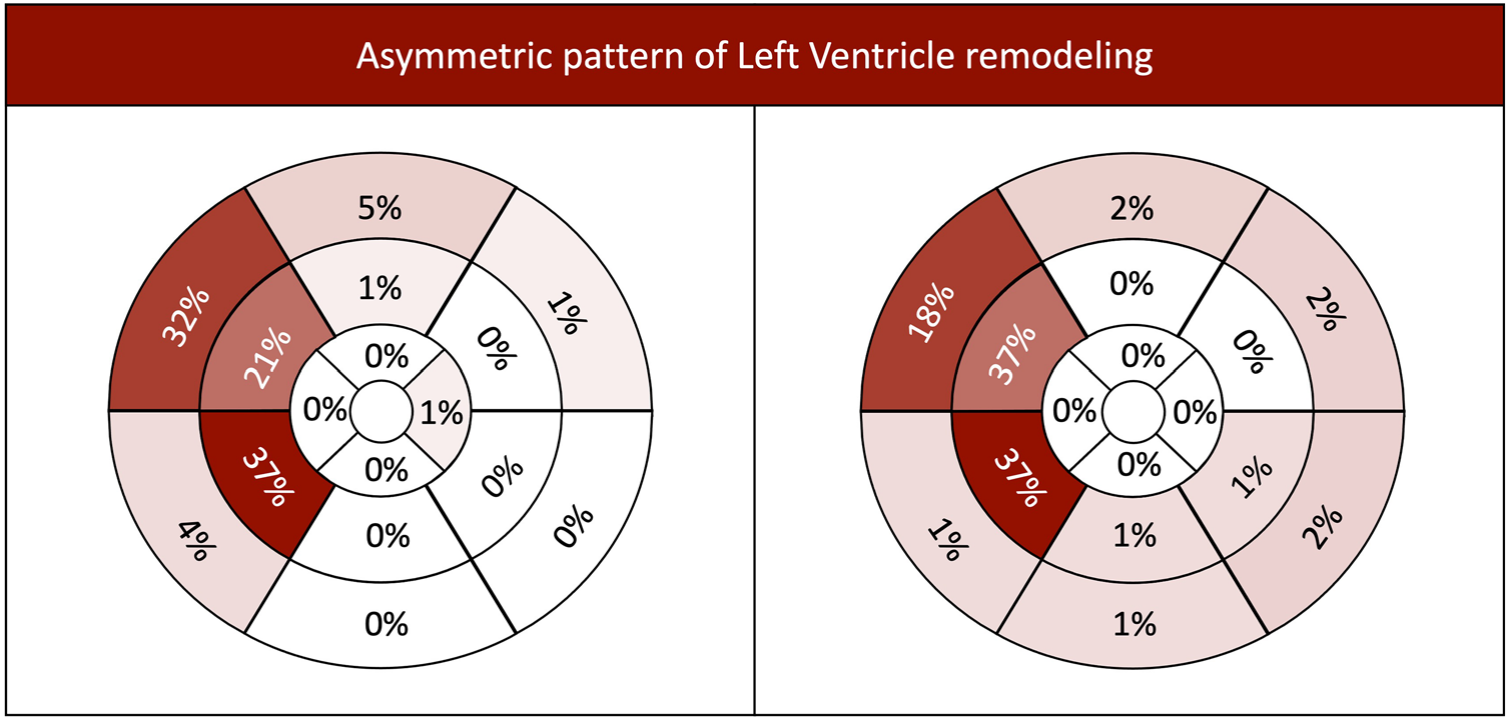
Distribution of hypertrophied LV segments in patients with asymmetric pattern of LV remodeling before (left) and after (right) AVR (based on 17-segment model of the LV).

Contrary to pre-operative predominant pattern of LV remodeling, 60% of the patients had a normal LV geometry after AVR, and this came from the adaptative change of patients with both concentric remodeling and concentric hypertrophy (Fig. 3). However, there was a similar percentage of patients with concentric remodeling after surgery when compared to preoperative data. This was the result of a reduction of LV mass in some patients with pre-operative concentric hypertrophy and notably from a change of pattern in some patients with normal LV geometry before surgery. After surgery, asymmetric wall thickening persisted in 40 patients (35% of patients with completed imaging evaluations), with a shift from basal to mid septum wall segments (Fig. 4).

We found no significant differences in the percentage of patients submitted to concomitant myectomy across distinct patterns of remodeling. However, this procedure was more frequently performed in patients with asymmetric hypertrophy (48% vs. 63%, p = 0.091).

## Discussion

This study describes the different morphological patterns of remodeling and LV hypertrophy in a cohort of patients with predominant normal flow, high gradient, preserved EF, severe AS. Our main findings were that: (1) LV response to chronic pressure overload differed widely between patients; (2) the severity of AS, functional repercussion and LV loading conditions were independently associated with the pattern of LV adaptation before surgery; (3) asymmetric wall thickening was highly prevalent (4) short-term follow up after surgical AVR revealed that LV response was dynamic after surgery, with diverse patterns of remodeling as well.

To our knowledge this is one of the few CMR studies specifically addressing LV remodeling and adaptation in patients with severe symptomatic AS both before and after surgical AVR. Previous works with CMR had smaller samples, included patients with moderate AS and did not provide longitudinal data following intervention.

As previously reported from echocardiographic studies and consistent in pathophysiological terms^8,23^, LV hypertrophy was prevalent, albeit far from being the sole pattern of LV adaptation. The significant proportion of patients with asymmetric hypertrophy may explain the relative proportion of distinct LV patterns in our study, more like that described by Dweck et al.^9^. LV mass estimation from TTE derives from linear M-mode measurements, which are greatly influenced by LV cavity dimension and also basal septal thickness. Basal septal thickness did not represent global LV wall thickening in a great proportion of patients as we report LV asymmetric thickening in more than half of them. This probably explains TTE mass overestimation. Distinct LV volumes by both techniques are also probably explained by differences in both TTE and CMR in what concerns spatial resolution and techniques for volume estimation: *Simpson´s* rule by TTE, with endocardial interface definition, probably affected by concentric remodeling and hypertrophy, *versus* each slice contour definition at short-axis segmentation by CMR. Contrary to ancillary echocardiographic studies relying on M-mode measurements and extrapolations, the estimation of LV mass and geometry by CMR more accurately reflects LV structural and geometrical changes. In the same scope, there is a proportion of patients with preoperative normal LV geometry and mass, and a predominant proportion of patients returning to this morphological pattern after AVR, which follows the expected physiological response after the treatment of a pressure overload. Nevertheless, some patients still maintain either concentric remodeling and hypertrophy after surgery or evolve from a normal LV geometry through concentric patterns. This was not certainly due to the procedure, as there were only two patients with moderate patient-prosthesis mismatch. As previously proposed^24,25^, this could be related to several factors, beyond valve disease, such as hypertension, neuro-endocrine environment, and their mutual interaction, precluding post-operative reverse remodelling. Additionally, the presence of replacement fibrosis, which was more prevalent in our patients with concentric hypertrophy and may represent an advanced stage of LV disease in this setting, might be a predictor of the impairment of reverse remodeling after AVR. Finally, genetic factors controlling extracellular matrix remodeling and protein/collagen deposition/turnover in the myocardium, may stem considerable differences on the phenotypic expression of the LV in this context^26,27^. Overall, we should stress out that at the first glance our cohort of patients is predominantly composed of a homogeneous group of patients: with the same phenotype of high gradient, normal flow, preserved EF severe AS. However, considerable heterogeneity exists as far as LV adaptation is concerned, and this falls in line with recent concepts emerging from observational studies^9^. Classically, the idea of progressive transition through LV phenotypes in patients with severe AS, from normal geometry to concentric remodeling, hypertrophy, eccentric hypertrophy, and adverse remodeling prevailed, and this would have implications towards the condition of flow and gradients^6^. Instead, it is becoming apparent that both initiation and inhibition of hypertrophy involve multiple distinct signalling pathways from the beginning of pressure overload, i.e., less than severe AS, supporting the multifactorial genesis of LV remodeling^2^. Indeed, the lack of relation between aortic valve orifice area and LV mass in our cohort supports this concept. Moreover, we found significant differences among distinct patterns of remodeling regarding LV function and loading. These findings are mirrored by the positive relation between LV mass and BNP values, and supposed to be related to the association between increased LV mass and LGE (both structural and tissue remodeling), as we previously demonstrated following additional correlation data from the same cohort^28^.Here we specifically excluded patients with exclusive junctional LGE, as there is also evidence that this pattern is highly prevalent, unspecific and unrelated to adverse prognosis^28,29^.

We also observed an asymmetric pattern of LV hypertrophy, as defined by CMR, in more than half of the patients. This is more than reported by Dweck et al.^9^ and higher above previously reported prevalence from surgical teams^30^, even considering that the definition was derived from pre-operative TTE in preceding studies, and not from CMR assessment. Asymmetric wall thickening was identified across distinct patterns of remodeling and was associated with higher transvalvular gradients, BNP levels and worse indexes of LV function. At CMR, this group was characterized by higher LVMi and absolute LGE mass. As previously proposed^31^ it would be tempting to interpret this as a genotypic predisposition, related to hypertrophic cardiomyopathy, to asymmetric rather than concentric remodeling in a pressure overload condition. However, this was observed in a high proportion of patients, well-above the estimated prevalence of any genetic cardiomyopathy, lacking additional morphologic and functional characteristics features. Besides, the presence of a pressure overload condition such as AS, seems counterintuitive regarding the concept of hypertrophic cardiomyopathy. Our belief is that genetics is far from being the sole main driver of such a common geometric pattern. Furthermore, we might also speculate that regional distribution of endocavitary pressure on basal LV segments, following Laplace´s law, could eventually explain asymmetric wall thickening in some particular segments in some of the patients. Additional studies focused on the possible interaction between genetics, clinical factors, haemodynamic conditions and original cavitary geometry before valve disease development, converging for asymmetric wall thickening, are needed. Ultimately, its clinical meaning and predictors are important to define as this could impact the decision of when to associate myectomy to surgical AVR.

## Limitations

Our study had some limitations. As it was conducted at a single tertiary centre with a moderate sample size, the size of the different pattern’s groups of LV remodeling is necessarily small, thus limiting the extrapolation of the results. Patients referred for transcatheter procedures were not included, this possibly excluding a significant proportion of patients with either eccentric or adverse patterns of remodelling.

Likewise, we were not able to collect accurate data specifically addressing the length of time from the diagnosis of severe AS, beginning of symptoms or clinical indication for aortic valve replacement. These would have been important when trying to understand the remodeling process. Similarly, we did not appropriately assess the impact of long-standing factors which might have also influenced LV adaptative response, such as both pre- and post-operative blood pressure and diabetes control. In the same way, we were not able to evaluate if surgical myectomy was independently related to the particular type of post-operative remodeling. As the design of our research protocol also included endomyocardial septal biopsies during AVR, we believe that this could explain the high prevalence of surgical myectomy reported by the surgical team. Unfortunately, we have no data addressing its extension, and this could have been important as to assess its impact on postoperative remodeling. However, we have recently published data on the same cohort showing that post-operative changes in LV mass and volumes were independent from surgical septal myectomy^32^.

From pre-operative CMR we did not have the suspicion of cardiac amyloidosis, but we could not definitely confirm this in the whole cohort as we did not perform additional targeted investigation^32^. Likewise, we were not able to exclude possible concomitant diagnosis of hypertrophic cardiomyopathy, eventually explaining the high prevalence of asymmetric LV hypertrophy.

Finally, this was mainly a descriptive imaging study, and our aim was not to find predictors for a specific type of remodeling. Unfortunately, we are also not yet able to provide prognostic data for distinct patterns of LV adaptation in the long-term follow-up after AVR.

## Conclusion

In this prospective cohort of patients with predominant classical severe symptomatic AS we have demonstrated that concentric hypertrophy was not the sole pattern of LV remodeling. Indeed, diverse patterns of adaptation exist, and asymmetric hypertrophy is a common finding, with distinct features in what concerns LV function and loading. LV re-adaptative remodeling following surgical AVR is also diverse and occurs in the short-term.

### Supplementary Information

Supplementary Information.

## Data Availability

All data generated or analysed during this study are included in this published article [and its supplementary information files].

## Abbreviations

ACE: Angiotensin-converting enzyme
ARBs: Angiotensin receptor blockers
AS: Aortic stenosis
AV: Aortic valve
AVA: Aortic valve area
AVmean: Mean aortic transvalvular pressure gradient
AVR: Aortic valve replacement
CMR: Cardiac magnetic resonance
ECG: Electrocardiography
EOA: Effective orifice area
GLS: Global longitudinal strain
hsTn-T: Cardiac troponin T
Htc: Hematocrit
LGE: Late gadolinium enhancement
LV: Left ventricle
LVEDVi: Left ventricular end-diastolic volume index
LVEF: Left ventricular ejection fraction
LVH: Left ventricular hypertrophy
LVMi: Left ventricular mass index
MRAs: Mineralocorticoid receptor antagonists
M/V: Geometric remodeling ratio (CMR)
NT-ProBNP: N-terminal pro b-type natriuretic peptide
NYHA: New York Heart Association
RWT: Relative wall thickness
TTE: Transthoracic echocardiogram

## References

[1] Vahanian A, Beyersdorf F, Praz F (2022). 2021 ESC/EACTS Guidelines for the management of valvular heart disease. Eur. Heart J..

[2] Abecasis J, Gomes Pinto D, Ramos S (2021). Left ventricular remodeling in degenerative aortic valve stenosis. Curr. Probl. Cardiol..

[3] Krayenbuehl, H. P., Hess, O. M., Monrad, E. S., *et al.* Clinical investigation left ventricular myocardial structure in aortic valve disease before, intermediate, and late after aortic valve replacement. 10.1161/01.cir.79.4.744 2522356

[4] Amanullah MR, Pio SM, Ng ACT (2021). Prognostic implications of associated cardiac abnormalities detected on echocardiography in patients with moderate aortic stenosis. JACC Cardiovasc Imaging.

[5] Ajmone Marsan N, Delgado V, Shah DJ (2022). Valvular heart disease: shifting the focus to the myocardium. Eur. Heart J..

[6] Badiani S, van Zalen J, Treibel TA (2016). Aortic stenosis, a left ventricular disease: insights from advanced imaging. Curr. Cardiol. Rep..

[7] Yarbrough WM, Mukherjee R, Ikonomidis JS (2012). Myocardial remodeling with aortic stenosis and after aortic valve replacement: Mechanisms and future prognostic implications. J. Thorac. Cardiovasc. Surg..

[8] Seiler C, Jenni R (1996). Severe aortic stenosis without left ventricular hypertrophy: Prevalence, predictors, and short-term follow up after aortic valve replacement. Heart.

[9] Dweck MR, Joshi S, Murigu T (2012). Left ventricular remodeling and hypertrophy in patients with aortic stenosis: Insights from cardiovascular magnetic resonance. J. Cardiovasc. Magn. Reson..

[10] Bohbot Y, Renard C, Manrique A (2020). Usefulness of cardiac magnetic resonance imaging in aortic stenosis. Circ. Cardiovasc. Imaging.

[11] Biederman RWW, Magovern JA, Grant SB (2011). LV reverse remodeling imparted by aortic valve replacement for severe aortic stenosis; is it durable? A cardiovascular MRI study sponsored by the American Heart Association. J. Cardiothorac. Surg..

[12] Mitchell C, Rahko PS, Blauwet LA (2019). Guidelines for performing a comprehensive transthoracic echocardiographic examination in adults: Recommendations from the American Society of Echocardiography. J. Am. Soc. Echocardiogr..

[13] Lang RM, Badano LP, Victor MA (2015). Recommendations for cardiac chamber quantification by echocardiography in adults: An update from the American Society of Echocardiography and the European Association of Cardiovascular Imaging. J. Am. Soc. Echocardiogr..

[14] Baumgartner H, Hung J, Bermejo J (2017). Recommendations on the echocardiographic assessment of aortic valve stenosis: A focused update from the European Association of Cardiovascular Imaging and the American Society of Echocardiography. Eur. Heart J. Cardiovasc. Imaging.

[15] Nagueh SF, Smiseth OA, Appleton CP (2016). Recommendations for the evaluation of left ventricular diastolic function by echocardiography: An update from the American Society of Echocardiography and the European Association of Cardiovascular Imaging. J. Am. Soc. Echocardiogr..

[16] Zoghbi WA, Chambers JB, Dumesnil JG (2009). Recommendations for evaluation of prosthetic valves with echocardiography and Doppler ultrasound. A report From the American Society of Echocardiography’s Guidelines and Standards Committee and the Task Force on Prosthetic Valves, Developed in Conjunction With the American College of Cardiology Cardiovascular Imaging Committee, Cardiac Imaging Committee of the American Heart Association. J. Am. Soc. Echocardiogr..

[17] Kramer CM, Barkhausen J, Flamm SD (2013). Standardized cardiovascular magnetic resonance (CMR) protocols 2013 update. J. Cardiovasc. Magn. Reson..

[18] Messroghli DR, Moon JC, Ferreira VM (2017). Clinical recommendations for cardiovascular magnetic resonance mapping of T1, T2, T2 and extracellular volume: A consensus statement by the Society for Cardiovascular Magnetic Resonance (SCMR) endorsed by the European Association for Cardiovascular Imaging (EACVI). J. Cardiovasc. Magn. Reson..

[19] Kawel-Boehm N, Hetzel SJ, Ambale-Venkatesh B (2020). Reference ranges (“normal values”) for cardiovascular magnetic resonance (CMR) in adults and children: 2020 update. J. Cardiovasc. Magn. Reson..

[20] Lamb, H. J., Beyerbacht, H. P., de Roos, A., *et al.* Left ventricular remodeling early after aortic valve replacement: differential effects on diastolic function in aortic valve stenosis and aortic regurgitation recommended articles mitral valve replacement after failed mitral ring insertion with or without leaflet/chordal repair for pure mitral regurgitation extracellular myocardial volume in patients with aortic stenosis predictor of left ventricular dysfunction after aortic valve replacement in mixed aortic valve disease (2002). 10.1016/s0735-1097(02)02604-9 12505232

[21] Ganau, A., Devereux, R. B., Roman, M. J., *et al.* Studies in hypertension patterns of left ventricular hypertrophy and geometric remodeling in essential hypertension. 10.1016/0735-1097(92)90617-v 1534335

[22] Cioffi, G., & Stefenelli, C. Comparison of left ventricular geometry and left atrial size and function in patients with aortic stenosis versus those with pure aortic regurgitation. 10.1016/s0002-9149(02)02563-8 12231084

[23] Cioffi G, Faggiano P, Vizzardi E (2011). Prognostic effect of inappropriately high left ventricular mass in asymptomatic severe aortic stenosis. Heart.

[24] Gavina C, Falcão-Pires I, Pinho P (2016). Relevance of residual left ventricular hypertrophy after surgery for isolated aortic stenosis. Eur. J. Cardio-thorac. Surg..

[25] Gavina C, Falcão-Pires I, Rodrigues J (2014). Load independent impairment of reverse remodeling after valve replacement in hypertensive aortic stenosis patients. Int. J. Cardiol..

[26] Rassi AN, Pibarot P, Elmariah S (2014). Left ventricular remodelling in aortic stenosis. Can. J. Cardiol..

[27] Pasipoularides A (2016). Calcific aortic valve disease: Part 2—Morphomechanical abnormalities, gene reexpression, and gender effects on ventricular hypertrophy and its reversibility. J. Cardiovasc. Transl. Res..

[28] Maltês S, Abecasis J, Santos RR (2023). LGE prevalence and patterns in severe aortic stenosis: When “junctional” means the same. Int. J. Cardiol..

[29] Yi JE, Park J, Lee HJ (2018). Prognostic implications of late gadolinium enhancement at the right ventricular insertion point in patients with non-ischemic dilated cardiomyopathy: A multicenter retrospective cohort study. PLoS ONE.

[30] Portuguesa De Cardiologia, R., Magro, P.L., & Sousa Uva, M. What is the role of septal myectomy in aortic stenosis? (2022). 10.1016/j.repc.2021.02.022 36062668

[31] NII-Electronic Library Service. 10.7748/nm.14.3.37.s16 27724148

[32] Abecasis J, Lopes P, Santos RR (2023). Prevalence and significance of relative apical sparing in aortic stenosis: insights from an echo and cardiovascular magnetic resonance study of patients referred for surgical aortic valve replacement. Eur. Heart J. Cardiovasc. Imaging.

