# Supplementary material for: Cardiac magnetic resonance patterns of left ventricular remodeling in patients with severe aortic stenosis referred to surgical aortic valve replacement
Source: Sci Rep. 2024 Mar 26;14:7085. doi: 10.1038/s41598-024-56838-0 (PMC10963734; doi:10.1038/s41598-024-56838-0)
Supplement: Supplementary file 1 — Supplementary Information. [file 41598_2024_56838_MOESM1_ESM.docx]

**SUPPLEMENTAL TABLES**

**Supplemental Table 1 –** CMR patterns of LV hypertrophy as previously defined by Dweck *et al*; normal values for CMR according to Kawel-Boehm *et al* [19].

**Supplemental Table 2 –** Comparative analyses between asymmetric and symmetric LV patterns before surgery.

|  | Asymmetric LV hypertrophy | Symmetric LV hypertrophy | *p-value* |
| --- | --- | --- | --- |
| Number of patients | 71 | 59 | - |
| Hypertension, n (%) | 56 (86) | 52 (91) | 0.380 |
| ACE inhibitors/ARBs, n (%) | 44 (62) | 40 (68) | 0.489 |
| Diuretics, n (%) | 25 (35) | 22 (37) | 0.808 |
| Beta-blockers, n (%) | 18 (25) | 20(34) | 0.286 |
| MRAs, n (%) | 1 (1) | 2 (3.4) | 0.592 |
| Diabetes mellitus, n (%) | 21 (48) | 13 (22) | 0.808 |
| Creatinine Clearance, ml/min | 71.8 ± 24.8 | 72.2± 20.8 | 0.907 |
| NT-pro BNP, pg/mL | 790 (303 - 2119.5) | 437 (145-1190) | **0.043** |
| Echocardiography | | | |
| Indexed Aortic Valve Area, cm^2^ | 0.39 ± 0.10 | 0.41 ± 0.08 | 0.434 |
| Mean Aortic gradient, mmHg | 64.2 ± 18.4 | 57.4 ± 16.1 | **0.028** |
| LV mass, g | 301.3 ± 101.9 | 263 ± 90.4 | **0.026** |
| LV indexed mass, g/m2 | 167.8 ± 56 | 146.9 ± 47.6 | **0.025** |
| LVEDV, mL | 89.8 ± 37.7 | 83.4 ± 28.6 | 0.148 |
| LVEDV indexed, mL/m^2^ | 49.2 ± 18.8 | 46.4 ± 14.7 | **0.347** |
| LV ejection fraction, % | 57.1 ± 10 | 59.1 ± 8.3 | 0.247 |
| GLS, % | -13.8 ± 3.6 | -15.8 ± 3.8 | **0.005** |
| Cardiac Magnetic Resonance | | | |
| Ejection Fraction, % | 57.9 ± 10 | 61.8 ± 9.8 | **0.028** |
| LV mass, g | 164.6 ± 55.3 | 127.8 ± 41.5 | **<0.001** |
| LV mass index, g/m^2^ | 90.6 ± 27.8 | 71.2 ± 21.1 | **<0.001** |
| LVEDV, mL | 163 ± 51.9 | 145.7 ± 39.7 | **0.038** |
| Indexed LVEDV, mL/m^2^ | 89.5 ± 24.6 | 81.2 ± 20.4 | **0.042** |
| Geometric remodeling, g/m^2^ | 1.1 (0.88 – 1.14) | 0.8 (0.77 – 0.97) | **<0.001** |
| LGE, g | 4.2 (0 – 11.75) | 1.9 (0 – 5.94) | **0.036** |
| Global native T1, ms | 1056 (1034-1072) | 1047 (1019-1071) | **0.599** |
| Global ECV, % | 23 (20-27) | 24 (21-26) | **0.631** |

Values are median (interquartile range), mean±standard deviation, ACE inhibitors - Angiotensin-converting-enzyme inhibitors, ARBs - Angiotensin receptor blockers, MRAs - Mineralocorticoid receptor antagonists, GLS – Global longitudinal strain , LV – left ventricle, LVEDV – left ventricle end diastolic volume, LGE – late gadolinium enhancement; **Bold** *P-*values are statistically significant; Patients with exclusive junctional LGE were excluded from the analyses of LGE.

NOTE: All data generated or analysed during this study are included in this published article [and its supplementary information files].
